# Impedance cardiography parameters reflecting left ventricular function are markers of pulmonary arterial hypertension

**DOI:** 10.3389/fcvm.2025.1526471

**Published:** 2025-05-07

**Authors:** Yang Wentao, Wang Yumiao, Yang Luanluan, Li Xin, Cui Xiaopei

**Affiliations:** ^1^ Department of Geriatric Medicine & Shandong Key Laboratory Cardiovascular Proteomics, Qilu Hospital of Shandong University, Jinan, Shandong, China; ^2^ Department of Emergency, Dongying People's Hospital, Dongying, China; ^3^ Department of Cardiology, Pingyin People's Hospital, Jinan, Shandong, China; ^4^ Department of Endocrinology and Metabolism, Dongying People's Hospital, Dongying, China

**Keywords:** pulmonary arterial hypertension, impedance cardiography, left ventricular stroke work, left ventricular stroke work index, prognosis

## Abstract

**Background:**

Pre-capillary pulmonary hypertension (PH) is characterized by pulmonary vascular remodeling and right heart failure. We aim to identify impedance cardiography (ICG) parameters with potential for pre-capillary PH screening and prognostic prediction.

**Methods:**

A discovery cohort consisting of 26 patients with pulmonary arterial hypertension (PAH) and 20 healthy volunteers was enrolled from August 2018 to March 2019. Another 100 patients who had undergone right heart catheterization (RHC) due to suspected PAH were enrolled from April 2019 to August 2020 as the validation cohort. In total, 62 patients with pre-capillary PH in the discovery and validation cohorts were followed up for 41 months. The relationships between ICG parameters and pre-capillary PH screening and prognostic prediction were studied.

**Results:**

Patients with pre-capillary PH exhibited lower left ventricular stroke work (LSW) and left ventricular stroke work index (LSWI) values compared to the healthy controls, which was further proved in the validation cohort [51.5 (41.8–67.2) vs. 69.7 (68.1–72.3) g·m/beat, *p* = 0.014 for LSW; 30.9 (26.5–40.9) vs. 41.7 (40.8–43.8) g·m/beat/m^2^, *p* = 0.026 for LSWI]. Patients with low risk status at baseline exhibited much higher LSW [57.1 (45.8, 73.1) vs. 45.8 (35.1, 57.4) g·m/beat, *p* = 0.002] and LSWI [35.1 (28.4, 43.7) vs. 27.2 (20.4, 36.3) g·m/beat/m^2^, *p* < 0.001] than those at intermediate/high risk. The cut-off points that predicted PAH low risk status were 57.85 g·m/beat (sensitivity 59% and specificity 63%) for LSW and 36.75 g·m/beat/m^2^ (sensitivity 61% and specificity 79%) for LSWI. During follow-up, the hazard ratio (HR) for a clinical worsening event in the LSW < 58 g·m/beat group was 8.80 [95% confidence interval (CI): 3.16–24.54; *p* = 0.0001]. This was the same in the LSWI < 37 g·m/beat/m^2^ group (HR = 7.36, 95% CI: 2.65–20.44; *p* = 0.0001).

**Conclusion:**

LSW and LSWI detected by ICG are useful in pre-capillary PH screening and valuable as long-term predictors of clinical worsening in pre-capillary PH treatment.

## Introduction

Pre-capillary pulmonary hypertension (PH), particularly pulmonary arterial hypertension (PAH), constitutes a severe cardiovascular condition that can lead to right heart failure or mortality. In response to elevated pulmonary arterial pressure, the right ventricle undergoes adaptive changes, which subsequently affect left ventricular function, a phenomenon referred to as ventricular interdependence. In patients with PAH, the left ventricle experiences reduced preload, compression due to an enlarged right ventricle, and pericardial constraints. Consequently, these patients exhibit decreased left ventricular (LV) end-diastolic volume, stroke volume (SV), and left ventricular free wall mass (1–3). The cross-sectional area of LV cardiomyocytes was significantly reduced in patients with PAH, accompanied by diminished contractile strength compared to healthy donors (2). While echocardiograms of patients with PAH demonstrate normal LV dimensions and ejection fraction, these patients exhibit LV mechanical dysfunction characterized by a reduced transmitral flow peak early diastolic (peak E) velocity, which is associated with poorer hemodynamics and outcomes (4, 5). Impedance cardiography (ICG) is a non-invasive and readily applicable technique for the simultaneous detection of lung ventilation and perfusion, based on changes in thoracic impedance. Several studies have already reported that ICG is comparable to the thermodilution method for cardiac output (CO) detection in patients with PH (4, 6). In addition to CO detection, there are ICG parameters that reflect left heart function. This study primarily assessed the utility of ICG parameters in evaluating left ventricular function for both screening and prognostic purposes in pre-capillary PH in comparison with right heart catheterization (RHC) parameters.

## Materials and methods

### Study design and participants

This study was conducted as an ambispective, cross-sectional investigation, having received approval from the institutional ethical review committee of Qilu Hospital of Shandong University (Approval ID: 2019-119). All patients at Qilu Hospital of Shandong University who were suspected of having PH and who underwent RHC from August 2018 to August 2020 were enrolled after obtaining written consent. The diagnosis of pre-capillary PH was established in accordance with current guidelines, characterized by a mean pulmonary arterial pressure (mPAP) > 20 mmHg, pulmonary vascular resistance (PVR) > 2 Wood Units (WU), and pulmonary arterial wedge pressure (PAWP) ≤ 15 mmHg, as determined by RHC (7). Patients diagnosed with idiopathic pulmonary arterial hypertension (IPAH), heritable PAH, drug-induced and toxin-induced PAH, PAH associated with congenital heart disease (CHD-PAH), portopulmonary hypertension (PoPH), PAH associated with connective tissue disease (CTD-PAH), and inoperable chronic thromboembolic pulmonary hypertension (CTEPH) were included in the study.

**Figure 1:**
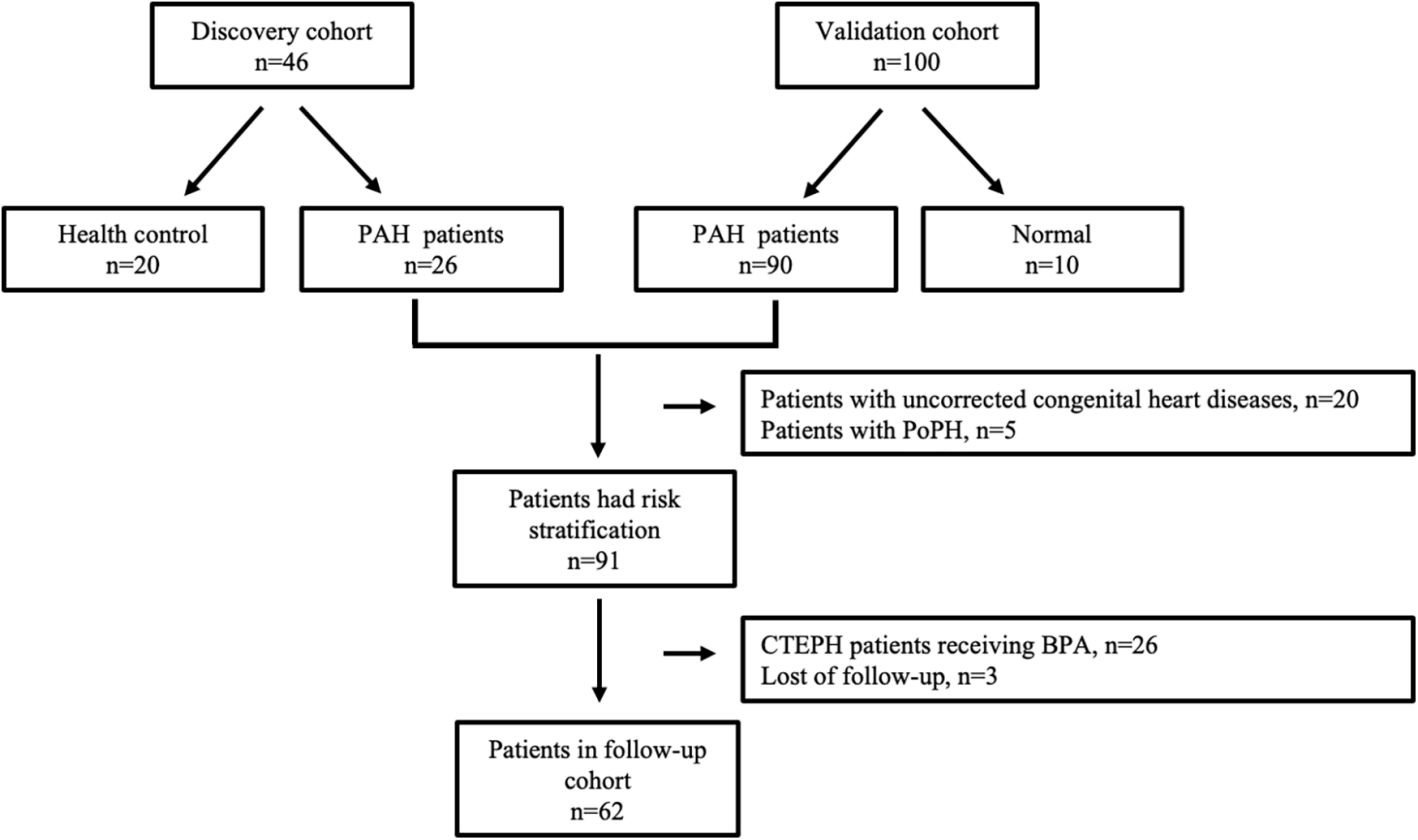
Flow chart of the study population.

Patients diagnosed with pre-capillary PH from August 2018 to March 2019, along with 20 healthy volunteers (HC group) exhibiting normal echocardiograms, were included in the discovery cohort. Patients enrolled from April 2019 to August 2020 constituted the validation cohort. Within the validation cohort, patients were categorized into the PAH group and the normal pressure (NP) group based on RHC results. All patients in both the discovery and validation cohorts were monitored periodically over a 41-month period, with the exception of the following: (1) patients with PAH with uncorrected congenital heart disease, including Eisenmenger syndrome; (2) patients with PAH due to lung diseases and/or hypoxia; (3) patients with PAH with unclear or multifactorial mechanisms; and (4) patients with CTEPH who had undergone thromboendarterectomy (PEA) or balloon pulmonary angioplasty (BPA). Only patients in the follow-up cohort underwent risk stratification. The flowchart detailing patient enrollment is presented in Figure 1.

## Procedures

### Clinical and laboratory parameters

Clinical data, including age, gender, height, weight, type of PAH, and all non-invasive parameters for risk stratification, were collected. These parameters encompass the World Health Organization functional class (WHO FC), 6 min walking distance (6MWD), N-terminal pro B-type natriuretic peptide (NT-proBNP), right atrial area (RAA) as assessed by echocardiography, right atrial pressure (RAP), mixed venous oxygen saturation (SvO_2_), and cardiac index (CI). Risk groups were categorized as low, intermediate, and high, corresponding to estimated 1-year mortality risks of <5%, 5%–20%, and >20%, respectively (7). The risk stratification model was proven to be usable in all patients with Group 1 PAH and CTEPH (8, 9).

### Right heart catheterization

A 7-Fr Swan-Ganz catheter (Edwards Lifesciences, California) was used for RHC. RAP, PAP, PAWP, SvO_2_, and CO by the thermodilution technique were recorded (10). PVR was calculated as (mPAP-PAWP)/CO. CI was calculated as CO/body surface area (BSA).

### Impedance cardiography

ICG measurements were carried out at rest before RHC by the same operator using a CSM3100 monitor (Shenzhen Qianfan Electronics Co. Ltd, China). ICG uses variations in the transthoracic impedance to a high-frequency (1,600 kHz), low-amperage (7 μA) alternating current across the thorax during cardiac ejection to calculate stroke volume. The left ventricular stroke work index (LSWI) calculation formula is as follows: LSWI = 0.0144 × (MAP − LAP) × SVI (11). Mean arterial pressure (MAP) was determined using the formula: MAP = (SAP + 2 × DAP)/3, where SAP represents systolic aortic pressure, and DAP denotes diastolic aortic pressure. The left arterial pressure (LAP) is a fixed value set at 7 Torr by the ICG system. The stroke volume index (SVI) was calculated as SV divided by BSA.

### Outcomes

During the 41-month follow-up period, clinical worsening events were recorded and adjudicated by independent experts in PH. These events were defined as follows: (1) all-cause mortality, (2) hospitalization due to worsening PAH, which includes non-elective hospitalization due to PAH or the initiation of parenteral prostanoid therapy, or (3) disease progression, characterized by a decrease in the 6MWD of ≥15% on two separate occasions, accompanied by either a deterioration in the WHO functional class, the necessity for new PAH-targeted medication, or the occurrence of decompensated right heart failure.

### Statistics

The normally distributed continuous variables are presented as mean ± standard deviation (SD), otherwise as median and interquartile (25%, 75%) ranges [M (Q1, Q3)]. Student’s *t*-test and the Mann–Whitney test were used for group comparison, as appropriate. The relationship between left ventricular stroke work (LSW)/LSWI and PAH was further analyzed by univariate and multivariate logistic regression. In multivariate logistic regression, we showed (1) unadjusted models and (2) model I, adjusted for covariates including age, sex, body weight index (BMI), and heart rate. Receiver operator characteristic (ROC) curves were used to determine the optimal diagnostic cut-off values of ICG parameters for PAH risk stratification. Cox proportional hazards regression was used to estimate hazard ratios (HRs) and 95% confidence intervals (CIs) of PAH clinical worsening risk in relation to LSW and LSWI. In the time-to-event analyses, end points were estimated with the use of the Kaplan–Meier method and were analyzed with the use of the log-rank test. Hazard ratios with 95% confidence intervals were estimated with the use of proportional-hazard models. The cut-off date of follow-up was 30 June 2023. A *p*-value < 0.05 was considered statistically significant. All analyses were performed using the statistical software packages R (http://www.R-project.org, The R Foundation) and EmpowerStats (http://www.empowerstats.com, X&Y Solutions, Inc., Boston, MA).

## Results

### Patients characteristics

In the discovery cohort, there were 26 patients with PAH, comprising 11 with IPAH, 6 with CHD-PAH, 2 with CTD-PAH, 6 with CTEPH, and 1 with PoPH. Additionally, 20 healthy controls were included. The median age of the participants was 29 years (interquartile range: 24–35 years), with 12 (60%) being female. The validation cohort consisted of 100 patients, among whom 10 subjects with mPAP ≤ 20 mmHg were categorized into the NP group. The remaining 90 subjects were classified into the PAH group, which included 24 (26.7%) with IPAH, 33 (36.6%) with CHD-PAH, 9 (10%) with CTD-PAH, and 20 (22.2%) with CTEPH, as detailed in Table 1.

**Table 1:** Clinical characteristics of the participants.

Variable	Discovery cohort	Validation cohort		
	(*n* = 26)	(*n* = 100)		
	PAH group	NP group	PAH/CTEPH group	*p*
		*n* = 10	*n* = 90	
Age [years, M (Q1, Q3)]	36.5 (29.0, 52.8)	33.0 (26.2, 41.8)	43.5 (33.0, 61.0)	0.060
Female [*n* (%)]	18 (69.2)	6 (60.0)	54 (60.0)	1.000
PAH etiology [*n* (%)]		–		
IPAH	11 (42.3)		24 (26.7)	
CHD-PAH	6 (23.1)		33 (36.6)	
Uncorrected	5		15	
Corrected	1		18	
CTD-PAH	2 (7.7)		9 (10)	
CTEPH	6 (23.1)		20 (22.2)	
PoPH	1 (3.8)		4 (4.4)	
Non-invasive metrics, M (Q1, Q3)				
WHO FC III/IV [*n* (%)]	11 (42.3)	0 (0.0)	40 (44.4)	<0.001
6MWD (m)	432 (366, 466)	482 (457, 521)	450 (405, 484)	0.364
NT-proBNP (pg/ml)	626 (151, 1,528)	152 (70.0–496)	311 (119–1,008)	0.315
RHC parameters, M (Q1, Q3)				
mPAP (mmHg)	59 (41, 65)	16 (14, 17)	40 (30, 56)	<0.001
PVR (Wood Units)	10.3 (5.6, 13.7)	1.4 (1.1, 1.5)	6.0 (3.3, 10.0)	<0.001
mRAP (mmHg)	6 (5, 7)	4 (3, 4)	5 (4, 8)	0.065
PAWP (mmHg)	10 (7, 12)	7 (6, 9)	8 (6, 10)	0.381
CI (L/min/m^2^)	2.8 (2.5, 3.3)	2.8 (2.4, 3.1)	2.7 (2.3, 3.0)	0.368

Healthy controls (*n* = 20): median age 29 (24, 35) years, female *n* = 12 (60%).

Continuous variables are presented as the mean with the standard deviation when distributed normally or otherwise as the median (lower quartile, upper quartile).

6MWD, 6 min walking distance; RHC, right heart catheterization; mPAP, mean pulmonary arterial pressure; PVR, pulmonary vascular resistance; mRAP, mean right atrial pressure; PAWP, pulmonary capillary wedge pressure; CI, cardiac index.

### LSW and LSWI are useful in PAH screening

As shown in Table 2, compared to health controls, patients with PAH had decreased LSW [58.7 (44.6, 71.6) vs. 81.2 (69.5, 100.9) g·m/beat, *p* < 0.001] and shorter left ventricular ejection time (LVET) [275 (240, 293) vs. 408 (333, 472) ms, *p* < 0.001]. A significant difference was also observed between the NP group and the PAH group in the validation cohort. LSW was 51.5 (41.8–67.2) g·m/beat in the PAH group compared to 69.7 (68.1–72.3) g·m/beat in the NP group (*p* = 0.014) and LSWI was 30.9 (26.5–40.9) vs. 41.7 (40.8–43.8) g·m/beat/m^2^ (*p* = 0.026).

**Table 2:** ICG parameters of the subjects.

Parameter	Discovery cohort			Validation cohort		
	(*n* = 46)			(*n* = 100)		
	Health control	PAH group	*P*	NP group	PAH/CTEPH group	*P*
	*n* = 20	*n* = 26		*n* = 10	*n* = 90	
CO [L/min, M (Q1, Q3)]	5.4 (4.6, 6.6)	4.3 (3.7, 4.9)	<0.001	5.5 (4.5–6.0)	4.0 (3.5, 5.0)	0.006
CI [L/min/m^2^, M (Q1, Q3)]	3.2 (2.7, 3.5)	2.6 (2.3, 3.2)	0.024	3.3 (2.8–3.5)	2.5 (2.0, 2.9)	0.009
SV [ml/beat, M (Q1, Q3)]	79 (67, 95)	58 (48, 70)	<0.001	69.5 (65.5–80.8)	55.0 (46.0–67.8)	0.015
SVI [ml/beat/m^2^, M (Q1, Q3)]	47.5 (38.5, 53.0)	37.0 (31.0, 42.0)	0.004	43.5 (40.2–45.8)	34.0 (29.0–40.0)	0.026
LSW [g·m/beat, M (Q1, Q3)]	81.2 (69.5, 100.9)	58.7 (44.6, 71.6)	<0.001	69.7 (68.1–72.3)	51.5 (41.8–67.2)	0.014
LSWI [g·m/beat/m^2^, M (Q1, Q3)]	46.4 (40.1, 57.9)	37.0 (27.7, 42.1)	<0.001	41.7 (40.8–43.8)	30.9 (26.5–40.9)	0.026
LVET [ms, M (Q1, Q3)]	408 (333, 472)	275 (240, 293)	<0.001	297.0 (279.5–345.0)	282.0 (228.5–352.0)	0.749
PEP [ms, M (Q1, Q3)]	74.0 (56.5, 113.8)	110 (100.0, 114.0)	0.466	104.0 (96.5–113.5)	103.0 (84.5–125.5)	0.616
UCG estimated sPAP	-	-	-	54.0 (34.0–56.0)	75.5 (58.0–88.8)	<0.001

CO, cardiac output; CI, cardiac index; SV, stroke volume; SVI, stroke volume index; LSW, left ventricular stroke work; LSWI, left ventricular stroke work index; LVET, left ventricular ejection time; PEP, left pre-ejection period; UCG, ultrasonic cardiogram; sPAP, systolic pulmonary arterial pressure.

### The relationship between LSW/LSWI and predicted PAH risk stratification

Patients were categorized into a low-risk group and an intermediate/high-risk group based on a comprehensive risk assessment for pulmonary arterial hypertension, as recommended by the 2022 European Society of Cardiology/European Respiratory Society (ESC/ERS) guidelines. As indicated in Table 3, patients classified as low risk at baseline demonstrated significantly higher LSW [57.1 (45.8, 73.1) vs. 45.8 (35.1, 57.4) g·m/beat, *p* = 0.002] and LSWI [35.1 (28.4, 43.7) vs. 27.2 (20.4, 36.3) g·m/beat/m^2^, *p* < 0.001] values than those at intermediate/high risk.

**Table 3:** Baseline characteristics of patients with PAH with different risk statuses.

Variable	Low risk	Intermediate/high risk	*p*
	*n* = 44	*n* = 47	
Age (years)	40 ± 14	54 ± 14	0.001
BMI (kg/m^2^)	22.6 ± 2.8	22.4 ± 2.8	0.762
LSW (g·m/beat)	57.1 (45.8, 73.1)	45.8 (35.1, 57.4)	0.002
LSWI (g·m/beat/m^2^)	35.1 (28.4, 43.7)	27.2 (20.4, 36.3)	<0.001
6MWD (m)	465 (435, 510)	405 (349,450)	<0.001
FC III/IV [*n* (%)]	11 (25)	33 (70)	<0.001
NT-proBNP (pg/ml)	154 (81, 234)	1,112 (321, 2,200)	<0.001
RHC parameters			
CO (L/min)	5.5 ± 1.0	4.3 ± 1.2	<0.001
CI (L/min/m^2^)	3.1 (2.9, 3.6)	2.7 (2.3, 2.9)	<0.001
mRAP (mmHg)	4.0 (3.0, 6.0)	6.0 (4.0, 9.8)	<0.001
mPAP (mmHg)	32.0 (28.0, 40.8)	55.5 (37.0, 64.5)	<0.001
PVR (Wood Units)	4.8 (3.3, 6.8)	10.1 (6.9, 13.3)	<0.001
SvO_2_ (%)	70.9 ± 5.4	61.0 ± 7.5	<0.001

Continuous variables are presented as the mean with the standard deviation when distributed normally or otherwise as the median (lower quartile, upper quartile).

BMI, body weight index; LSW, left ventricular stroke work; LSWI, left ventricular stroke work index; 6MWD, 6 min walking distance; RHC, right heart catheterization; CO, cardiac output; CI, cardiac index; mRAP, mean right atrial pressure; mPAP, mean pulmonary arterial pressure; PVR, pulmonary vascular resistance; SvO_2_, venous oxygen saturation.

**Figure 2:**
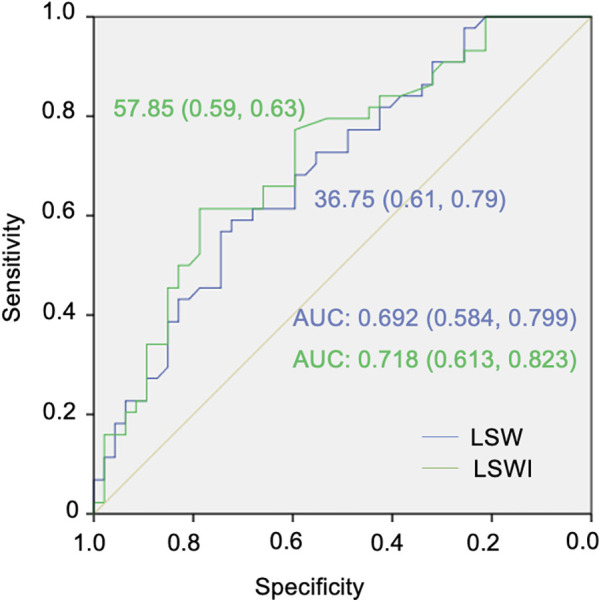
Receiver operating characteristic (ROC) curves for the cut-off points for left ventricular stroke work (LSW) and left ventricular stroke work index (LSWI) in pulmonary arterial hypertension (PAH) low-risk status classification.

The ROC curve depicted in Figure 2 demonstrates that the areas for LSW and LSWI were significant in predicting low-risk status for PAH. The area under the curve (AUC) was 0.692 (95% CI 0.584–0.799) for LSW and 0.718 (95% CI 0.613–0.823) for LSWI. The cut-off points for predicting low-risk status for PAH, determined using the Youden index, were 57.85 g·m/beat for LSW (sensitivity 59% and specificity 63%) and 36.75 g·m/beat/m² for LSWI (sensitivity 61% and specificity 79%), as illustrated in Figure 2.

### The relationship between LSW/LSWI and clinical worsening events

The primary endpoint was a composite of clinical worsening events, encompassing all-cause mortality, hospitalization due to worsening PAH, or disease progression. A cohort of 62 patients was monitored over a median duration of 41 months, during which one participant passed away, and 14 patients experienced disease progression. A one-unit increase in the natural log-transformed LSW was associated with an 11.5% reduction in the risk of PAH clinical worsening (HR = 0.885, 95% CI: 0.839–0.933). Additionally, there was a 21.1% reduction in PAH clinical worsening for such an increase in LSWI (HR = 0.789, 95% CI: 0.712–0.875). Following multivariable adjustment, elevated LSW (HR = 0.859, 95% CI: 0.797–0.926) and LSWI (HR = 0.795, 95% CI: 0.713–0.886) remained significantly associated with a reduced incidence of PAH clinical worsening events (Table 4).

**Table 4:** Relationship between per unit increment in natural log-transformed LSW and LSWI and clinical worsening events during follow-up.

Variable	Non-adjusted model		Model I	
	HR (95% CI)	*p*	HR (95% CI)	*p*
LSW (g·m/beat)	0.885 (0.839, 0.933)	<0.001	0.859 (0.797, 0.926)	<0.001
LSWI (g·m/beat/m^2^)	0.789 (0.712, 0.875)	<0.001	0.795 (0.713, 0.886)	<0.001

Model I was adjusted for potential confounding factors, including age, sex, BMI, and heart rate.

LSW, left ventricular stroke work; LSWI, left ventricular stroke work index.

**Figure 3:**
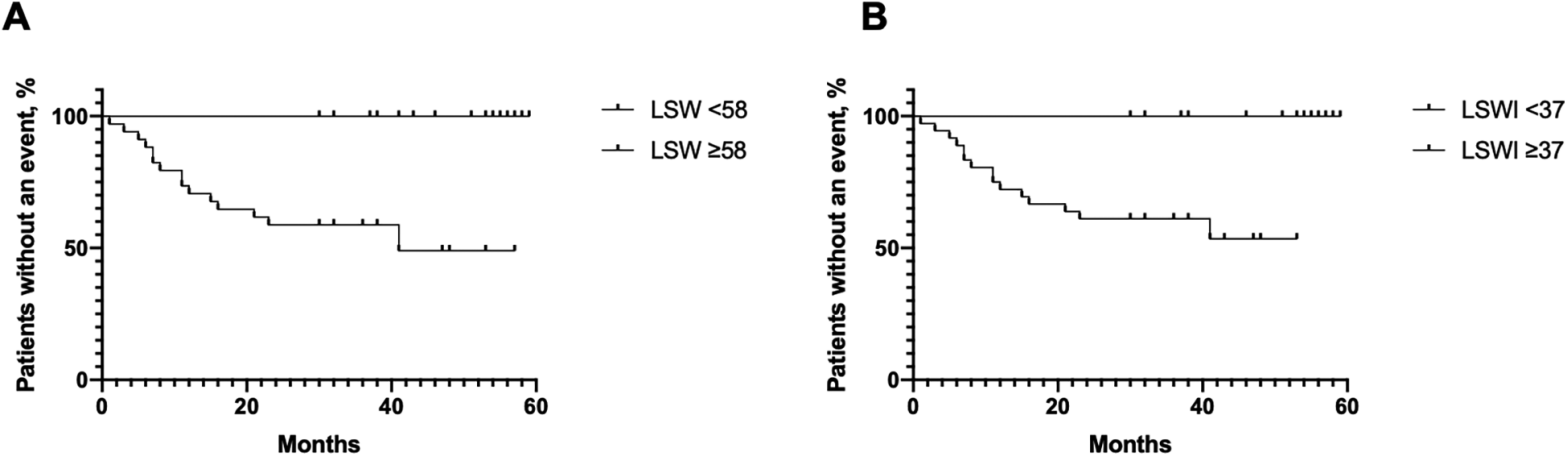
Kaplan–Meier curves for clinical worsening events related to pulmonary arterial hypertension (PAH) worsening in patients with low and high left ventricular stroke work (LSW) **(A)** and left ventricular stroke work index (LSWI) **(B).**

In total, 34 patients exhibited an LSW of less than 58 g·m/beat, among whom 15 patients (44.1%) experienced a clinical worsening event during the follow-up period. In contrast, no clinical worsening events were observed in patients with an LSW of 58 g·m/beat/m^2^ or greater. Similarly, 36 patients had an LSWI of less than 37 g·m/beat/m^2^, with 15 patients (41.7%) experiencing a clinical worsening event during follow-up, compared to none in the cohort with an LSWI of 37 g·m/beat/m^2^ or greater. The hazard ratio for a clinical worsening event in the group with LSW less than 58 g·m/beat was 8.80 (95% CI: 3.16–24.54; *p* = 0.0001), which is consistent with the LSWI less than 37 g·m/beat/m^2^ group (HR = 7.36, 95% CI: 2.65–20.44; *p* = 0.0001) (Figure 3).

## Discussion

PAH is a progressive and debilitating condition that ultimately leads to right heart failure and mortality. Despite advancements in PAH-specific therapies, which have improved the 5-year survival rate from 34% in 1991 to 71.2% in 2019 (12), the outcomes remain unsatisfactory, particularly as the majority of patients with PAH are young individuals of childbearing age. The GRIPHON study demonstrated that patients with a disease duration of less than 6 months derive greater benefit from PAH-specific therapies (13), underscoring the critical importance of early screening and diagnosis. Echocardiography, electrocardiography, and computed tomography are established methods for PAH screening (14–16). In this study, we have identified ICG as a novel and effective method for PAH screening. Previously, ICG was primarily utilized in pulmonary hypertension to assess cardiac output, with several studies confirming a moderate to strong correlation between ICG-detected cardiac output and RHC-detected cardiac output (17–19). Our current research demonstrates that LSW and LSWI detected by ICG are valuable parameters for PAH screening and risk stratification. PAH is a condition characterized by significant dilation of the right heart chamber, leading to impaired RV contractility (20–22). Emerging evidence suggests that right ventricular remodeling also impacts left ventricular function in PH, a phenomenon known as ventricular interdependence (23, 24). Both the right and left ventricles are enclosed within the pericardium and share the septum and myocardial fibers. Consequently, overload of the right chamber affects left ventricular function, which in turn influences the right ventricle (5, 25). In severe PH, the ventricular septum becomes flattened, imparting a characteristic D shape to the left ventricle (26). Cardiac magnetic resonance imaging (MRI) has demonstrated that left ventricular end-diastolic volume, stroke volume, and ejection fraction are reduced in patients with PAH (1, 3), accompanied by left ventricular myocardial fibrosis and atrophy (27). In a rat model of right ventricular failure secondary to CTEPH, left ventricular free wall mass decreased due to myocyte shrinkage, an adaptive atrophic remodeling response to right ventricular hypertrophy (28). Left ventricular myocyte atrophy has also been confirmed in patients with PAH who underwent left ventricular endomyocardial biopsy (2). There is accumulating evidence that left ventricular parameters are valuable for both PAH screening and prognosis prediction (29). Lindholm et al. found that low left ventricular peak global longitudinal strain (GLS) is indicative of increased mPAP and PVR (30). LV systolic strain (31), peak E velocity (32), LV E/e′, LV filling time (33), the ratio of RV end-diastolic area to LV end-diastolic area (34), and LV longitudinal strain (Ell LV) (35) have all been identified as predictors of mortality in PAH. These parameters are obtained from echocardiography or MRI, both of which require specialized techniques and equipment.

In cardiac physiology, the external work (EW) performed by the LV during a single heartbeat is denoted by LSW. The Frank–Starling law is characterized by a ventricular function curve indicating that LSW increases with left ventricular end-diastolic pressure (LVEDP) (36). Additionally, there exists a positive correlation between the myocardial energetic efficiency index (MEEi) and LSW. Both low LVEDP and MEEi are predictive of short- and long-term cardiovascular events (37). In the present study, we employed ICG to measure LSW and LSWI, demonstrating that these parameters are valuable for both pre-capillary PH screening and risk stratification. ICG is a non-invasive and easily applicable method, offering a more cost-effective alternative to echocardiography or MRI in clinical practice. ICG is recommended for specific populations at high risk for PAH, including patients with connective tissue diseases, individuals with congenital heart defects who have undergone repair, and subjects with hereditary predispositions to PAH.

Several parameters are incorporated into PAH risk stratification based on estimated 1-year mortality (7). Currently, numerous PAH-targeted drugs are in use, with the primary treatment objective being the attainment of a low-risk status. RV failure is a significant determinant of symptoms and reduced survival in PAH (38). In the present study, we initially demonstrated that LSW/LSWI is correlated with baseline risk status, with patients in the intermediate/high-risk categories at baseline exhibiting lower LSW/LSWI. Furthermore, we established that lower LSW/LSWI is associated with a higher rate of clinical worsening during follow-up, even with double or triple combinations of PAH-targeted drugs. Low LSW/LSWI indirectly reflects impaired RV contractility due to ventricular interdependence and may hold substantial clinical relevance.

## Limitations

First, our cohort was derived from a single center and was characterized by a limited sample size. Second, ICG was conducted solely at baseline, without dynamic monitoring during the follow-up period. Therefore, a future multi-center prospective cohort study with continuous ICG detection throughout the follow-up is warranted.

## Conclusion

LSW and LSWI, as detected by ICG, are instrumental in the screening of pre-capillary PH and serve as significant long-term predictors of clinical deterioration in the management of PAH.

## Data Availability

The original contributions presented in the study are included in the article/Supplementary Material, further inquiries can be directed to the corresponding authors.
